# COVID-19 and coinfection with *Clostridioides* ( *Clostridium* ) *difficile* in an infant with gastrointestinal manifestation

**DOI:** 10.31744/einstein_journal/2020RC6048

**Published:** 2020-11-25

**Authors:** Jane Oba, Clovis Artur Silva, Ricardo Katsuya Toma, Werther Brunow de Carvalho, Artur Figueiredo Delgado

**Affiliations:** 1 Hospital Israelita Albert Einstein São PauloSP Brazil Hospital Israelita Albert Einstein, São Paulo, SP, Brazil.; 2 Universidade de São Paulo Hospital das Clínicas Faculdade de Medicina São PauloSP Brazil Instituto da Criança, Hospital das Clínicas, Faculdade de Medicina, Universidade de São Paulo, São Paulo, SP, Brazil.

**Keywords:** COVID-19, Coronavirus infections, Coinfection, Clostridium, Gastrointestinal tract, Infant

## Abstract

We report the clinical case of an infant with severe acute respiratory syndrome coronavirus 2 (SARS-CoV-2) infection with gastrointestinal signs and symptoms, predominantly vomiting. The patient also had colic, poor feeding, mild diarrhea and mild rhinorrhea without fever. The child had evidence of altered coagulation, increased interleukin 10, moderate dehydration and she was admitted to the pediatric intensive care unit. Simultaneously, the patient was diagnosed as *Clostridioides difficile* infection, which possibly may have facilitated the persistence of SARS-CoV-2 in feces, for more than 27 days, even after the nasopharyngeal test turned negative. This coinfection might exacerbate the gastrointestinal signs and symptoms and increased the possibility of fecal-oral transmission of SARS-CoV-2 and *Clostridioides* . The patient was breastfed and received complementary infant formula, hydrated with intravenous fluid, and was discharged without complications, 4 days after admission.

## INTRODUCTION

The novel coronavirus disease (COVID-19) caused by severe acute respiratory syndrome-related coronavirus 2 (SARS-CoV-2) rapidly spread as a global pandemic. Emerging data regarding the clinical characteristics of infected children from China, Europe and the United States have shown that almost 20% of infection occurred in infants (less than 12 months), and the severity of illness is higher compared with older children.^(^^1^^–^^3^^)^ In addition, the potential harm of COVID-19 in infants and its prognosis remain unknown. The infection in infants have distinct clinical/radiologic characteristics from adults, and accounts for 48% of hospitalizations of all infected children and adolescents.^(^^2^^)^ Reported clinical manifestations among infants with SARS-CoV-2 infection are predominantly respiratory, such as fever, cough, rhinorrhea, increased work of breathing and lethargy. Moreover, almost 25% of cases also include gastrointestinal (GI) events, like vomiting, diarrhea, abdominal pain, feeding intolerance or decreased intake.^(^^1^^,^^2^^)^

Nearly 50% of infected children with SARS-CoV-2 were also coinfected with respiratory pathogens.^(^^2^^,^^4^^)^ Attention now is given to the GI tract,^(^^5^^)^ since recent evidence has suggested that SARS-CoV-2 can actively colonize and replicate in the GI tract.^(^^1^^,^^6^^)^ These findings have important implications for disease transmission and management, and the occurrence of enteropathogenic coinfection, particularly *Clostridioides difficile* (CD), formerly known as *Clostridium difficile* .

Infants colonized with toxigenic *C. difficile* rarely develop clinical disease, and the reasons are unknown.^(^^7^^)^ There are substantial gaps in the available data regarding COVID-19 in infants and coinfection with *C. difficile* . We describe a case report of an infant with COVID-19 coinfected with *C. difficile* . To the best of our knowledge, there are no reports about this kind of enteropathogenic coinfection in neonates and infants.

## CASE REPORT

**Figure 1:**
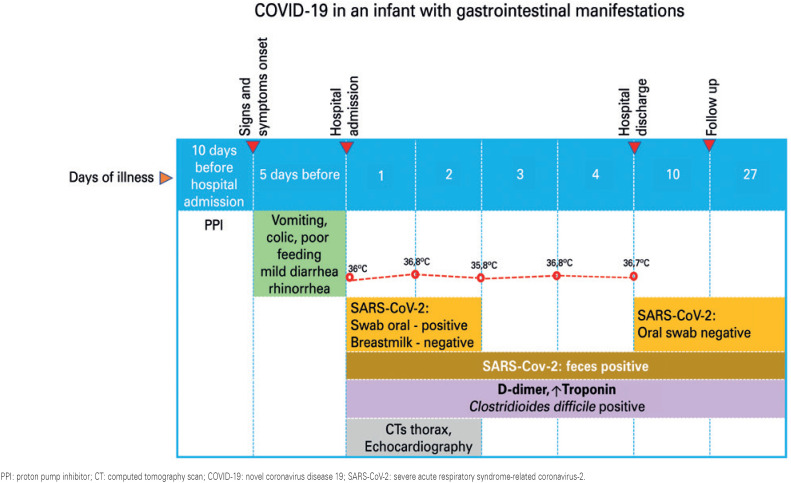
Chronology of clinical, laboratory and imaging events

A 2-month-old female infant previously healthy, presented to the pediatric emergency department. The infant's mother reported weight loss, colic, crying for feeding alternating with lethargy, mild diarrhea and worsening of vomiting beginning 5 days before ( Figure 1 ). Previously, she reported nasal congestion, sporadic cough, conjunctivitis, small skin vesicles on the chest and no fever. The real time reverse transcription-polymerase chain reaction (RT-PCR) SARS-CoV-2 nasopharyngeal test result was positive ( Table 1 ). The mother had been infected with SARS-CoV-2 10 days before the child. On physical examination, the infant was markedly irritable alternating with lethargy, with painful facies suggestive of abdominal colic associated to interruption of feeding several times, moderate dehydration, and hypothermia (<36°C). Her weight was 4.8kg, she had a mottled skin, prolonged capillary refill and a decreased urine output. Pulse rate was 125 beats/minute, respiratory rate was 54 breaths/minute, and oxygen saturation by pulse oximetry of 97% in room air. She was transferred to the pediatric intensive care unit (PICU) after fluid resuscitation.

**Table 1:** Laboratory and imaging results during the clinical course

Variables	Hospital admission	Day 10	Day 27
SARS-CoV-2	Oral swab: positive Stool: positive		Oral swab: negative Stool: positive
Hemoglobin, g/dL	11.4	9.6	10.5
Hematocrit, %	31.9	27.7	30.5
White blood cell count, μL	12,460	9,940	7,970
Neutrophils, μL (1000-8500)	623	2,565	1,363
Lymphocyte, μL (4000-13500)	9,843	6,706	6,481
Platelets, ×10^3^/μL (150-450)	470	503	427
CRP, mg/L (<5.0)	<1	0.3	<0.3
Albumin, g/dL (3.1-5)	4.1		
Ferritin, ng/mL (8.46-580)	503	388	
AST, U/L (0-32)	70		36
ALT, U/L (<25)	42		18
Urea, mg/dL (4-16)	8		
Creatinine, mg/dL	0.30		
Prothrombin time, (16-26.1)	29.6		27.9
Fibrinogen, mg/dL (200-400)	177		161
TTPa, (25.6-35.5)			33.5
D-dimer, ug/mL (<500)	641	751	
Troponin, pg/mL (<5)	11	22	16
IL-6, pg/nL (1.5-7.0)	2.2		
IL-10, pg/nL (0-2)	2.3		
Hemoculture	Negative		
*Clostridioides difficile* in stool	Positive	Positive	Positive
Echocardiography	Normal	Normal	
Transfontanellar brain ultrasonography			Normal
Abdominal ultrasonography	Normal		

SARS-CoV-2: severe acute respiratory syndrome-related coronavirus-2. CRP: C-reactive protein; AST: aspartate transaminase; ALT: alanine aminotransferase; IL: interleukine.

The patient was born at term, had no pre-existing medical conditions or use of antibiotics, and had been breastfed and complemented with infant formula since the second week of life.

Table 1 summarizes data on blood biochemistry, urine, stool analysis, coagulation tests and infection biomarker tests performed after confirming SARS-CoV-2 infection by RT-PCR upon admission, and in follow-up. Serology for SARS-CoV-2 was IgM positive (2.11AU/mL, with normal range <0.90) and IgG was negative. The SARS-CoV-2 PCR persisted positive in fecal samples for more than 27 days, although the respiratory tract test was negative. Additional viral panel testing, urine and fecal culture were negative, and only the nucleic acid amplification test (NAAT or PCR) was positive for *C. difficile* .

The helical multislice chest computed tomography (CT) scan, Doppler echocardiography and abdominal ultrasonography were normal.

During 4 days of hospitalization in the PICU, the patient persisted with abdominal colic, and required additional intravenous fluids due poor acceptance of breast milk or infant formula. The proton pump inhibitor initially prescribed was withdrawn. The patient was not prescribed antivirals, glycocorticoids or antimicrobials.

## DISCUSSION

The clinical reports of COVID-19 in infants reveal that GI symptoms might be the sole clinical presentation of the disease, more prominent than respiratory symptoms, like the patient described.^(^^2^^,^^3^^,^^5^^)^ It is important to highlight that COVID-19 in infants can be a severe illness compared with older children due to immaturity of the immune system. Consequently infants are potentially at risk for more significant complications and might require ICU support.^(^^2^^,^^8^^)^

Coinfections with others respiratory pathogens than SARS-CoV-2 are common, but with enteropathogens, particularly with *C. difficile* , have not been reported yet in infants. The clinical spectrum of *C. difficile* disease ranges from self-limiting secretory diarrhea to pseudomembranous colitis and septic shock. Two important features of *C. difficile* in infants are the highest incidence in the first year of life (14% to 37%) and frequent colonization with no clinically relevant symptoms.^(^^7^^)^ Polymerase chain reaction assay *C. difficile* detects the regulatory gene (tcdC) responsible for production of toxins A and B, with no detection of toxin, and the results often reflect *C. difficile* colonization rather than disease. For reasons that remain undefined, the colonized infants show no toxigenic effects from exotoxins released by *C. difficile* , in contrast to older children and adults who are susceptible to severe diarrhea and colitis. It has been proposed that the immature intestinal mucosa might lack receptors for *C. difficile* toxin.^(^^7^^)^ Other important factor is the protective properties of breastmilk, since breastfed infants have lower rates of *C. difficile* colonization (14%) compared to formula-fed infants (30%). In this case, damage to the gut due to SARS-CoV-2 might facilitate the coinfection with *C. difficile* ; and despite the protective factors of breast milk and the infant immunity, these were not enough to cease the elimination of the virus by feces. Additionally, the acid-suppression with proton pump inhibitor might facilitate the infection with *C. difficile* .^(^^9^^)^

The patient's laboratory findings showed minor changes in white blood cell counts and the inflammatory markers, C-reactive protein and ferritin were normal ( Table 1 ), as reported by other similar study.^(^^3^^)^ Nevertheless, more specific biomarkers, such as altered coagulation screen, thrombocytosis, increased IL-10 serum concentration and D-dimer suggested any degree of a systemic inflammatory disease. The Doppler echocardiography and chest CT of patient were normal. The importance of CT to diagnose COVID-19 in children is debated by some authors, because one-half of the cases did not show any radiologic changes during course of the disease.^(^^4^^)^

The patient was not prescribed antivirals, glycocorticoids, or antimicrobial agents considering the efficacy of these drugs is uncertain.^(^^10^^)^ We encouraged breastfeeding because it is considered protective for SARS-CoV-2 and *C. difficile* , and is an unlikely source of infection transmission. In addition, there is insufficient data to demonstrate efficacy of probiotics in treating *C. difficile* infection.

In agreement with other authors, the stool test remained positive for SARS-CoV-2 1 month after diagnosis, suggesting the possibility of prolonged fecal-oral transmission.^(^^4^^)^

## CONCLUSION

This case shows the long-term impact of COVID-19 on infant's health is still unknown. Many uncertainties persist regarding the infection by SARS-CoV-2 in infants, mainly when coinfected by other enteropathogens. More robust data and studies with a larger sample of infants are warranted to clarify the determinants of gastrointestinal infection presentation and severity. Breastfeeding seems to be a protection factor and may be continued in mothers with SARS-CoV-2.

## References

[1] 1. Dong Y, Mo X, Hu Y, Qi X, Jiang F, Jiang Z, et al. Epidemiology of COVID-19 among children in China. Pediatrics. 2020;145(6):e20200702. 10.1542/peds.2020-0702 32179660

[2] 2. Götzinger F, Santiago-García B, Noguera-Julián A, Lanaspa M, Lancella L, Calò Carducci FI, Gabrovska N, Velizarova S, Prunk P, Osterman V, Krivec U, Lo Vecchio A, Shingadia D, Soriano-Arandes A, Melendo S, Lanari M, Pierantoni L, Wagner N, L’Huillier AG, Heininger U, Ritz N, Bandi S, Krajcar N, Roglić S, Santos M, Christiaens C, Creuven M, Buonsenso D, Welch SB, Bogyi M, Brinkmann F, Tebruegge M; ptbnet COVID-19 Study Group. COVID-19 in children and adolescents in Europe: a multinational, multicentre cohort study. Lancet Child Adolesc Health. 2020;4(9):653-61. 10.1016/S2352-4642(20)30177-2 PMC7316447 32593339

[3] 3. Feld L, Belfer J, Kabra R, Goenka P, Rai S, Moriarty S, et al. A case series of the 2019 novel coronavirus (SARS-CoV-2) in 3 febrile infants in New York. Pediatrics. 2020;146(1):e20201056. 10.1542/peds.2020-1056 32404431

[4] 4. Wu Q, Xing Y, Shi L, Li W, Gao Y, Pan S, et al. Coinfection and other clinical characteristics of COVID-19 in children. Pediatrics. 2020;146(1):e20200961. 10.1542/peds.2020-0961 32376725

[5] 5. Oba J, Carvalho WB, Silva CA, Delgado AF. Gastrointestinal manifestations and nutritional therapy during COVID-19 pandemic: a practical guide for pediatricians. einstein (São Paulo). 2020;18:eRW5774. Review. 10.31744/einstein_journal/2020RW5774 PMC7346091 32667418

[6] 6. Tian Y, Rong L, Nian W, He Y. Review article: gastrointestinal features in COVID-19 and the possibility of faecal transmission. Aliment Pharmacol Ther. 2020;51(9):843-51. Review. 10.1111/apt.15731 PMC7161803 32222988

[7] 7. Schutze GE, Willoughby RE; Committee on Infectious Diseases; American Academy of Pediatrics. Clostridium difficile infection in infants and children. Pediatrics. 2013;131(1):196-200. Review. 10.1542/peds.2012-2992 23277317

[8] 8. McLaren SH, Dayan PS, Fenster DB, Ochs JB, Vindas MT, Bugaighis MN, et al. Novel coronavirus infection in febrile infants aged 60 days and younger. Pediatrics. 2020; 146(3):e20201550. 10.1542/peds.2020-1550 32527752

[9] 9. Borali E, De Giacomo C. Clostridium difficile infection in children: a review. J Pediatr Gastroenterol Nutr. 2016;63(6):e130-40. Review. 10.1097/MPG.0000000000001264 27182626

[10] 10. Hong H, Wang Y, Chung HT, Chen CJ. Clinical characteristics of novel coronavirus disease 2019 (COVID-19) in newborns, infants and children. Pediatr Neonatol. 2020;61(2):131-2. 10.1016/j.pedneo.2020.03.001 PMC7129773 32199864

