# Supplementary material for: COVID-19 and coinfection with Clostridioides ( Clostridium ) difficile in an infant with gastrointestinal manifestation
Source: Einstein (Sao Paulo). 2020 Nov 25;18:eRC6048. doi: 10.31744/einstein_journal/2020RC6048 (PMC7690928; doi:10.31744/einstein_journal/2020RC6048)
Supplement: Translation

# Translation: COVID-19 e coinfecção por *Clostridioides* ( *Clostridium* ) *difficile* em lactente com manifestações gastrintestinais

**Authors:** Jane Oba, Clovis Artur Silva, Ricardo Katsuya Toma, Werther Brunow de Carvalho, Artur Figueiredo Delgado

**Affiliations:** 1 São PauloSP Brasil Hospital Israelita Albert Einstein, São Paulo, SP, Brasil.; 2 São PauloSP Brasil Instituto da Criança, Hospital das Clínicas, Faculdade de Medicina, Universidade de São Paulo, São Paulo, SP, Brasil.

**Keywords:** COVID-19, Infecções por coronavírus, Coinfecção, Clostridium, Trato gastrointestinal, Lactente

## Abstract

Relatamos o caso clínico de uma lactente com infecção por coronavírus da síndrome respiratória aguda grave 2 (SARS-CoV-2) com sinais e sintomas gastrintestinais – predominantemente vômitos. A paciente apresentou, adicionalmente, cólica, dificuldade para mamar, evacuações amolecidas e rinorreia leve, sem febre. Houve evidências de alterações da coagulação, aumento de interleucina 10 e desidratação moderada, que justificaram internação na unidade de terapia intensiva. Simultaneamente, a paciente foi diagnosticada com infecção por *Clostridioides difficile* , que pode ter facilitado a persistência do SARS-CoV-2 nas fezes por mais de 27 dias, mesmo após negativação do teste nasofaríngeo. Essa coinfecção pode ter exacerbado os sinais e sintomas gastrintestinais e aumentado a possibilidade da transmissão do SARS-CoV-2 e *Clostridioides* . A paciente foi mantida em aleitamento materno e complemento com fórmula infantil, recebeu hidratação intravenosa e teve alta hospitalar, sem complicações, após 4 dias de internação.

## INTRODUÇÃO

A doença pelo novo coronavírus 2019 (COVID-19), causada pelo coronavírus da síndrome respiratória aguda grave 2 (SARS-CoV-2), espalhou-se rapidamente como uma pandemia global. Dados emergentes sobre as características clínicas de crianças infectadas da China, da Europa e dos Estados Unidos mostraram que quase 20% das infecções ocorreram em lactentes (abaixo de 12 meses de idade) e, nesse caso, a gravidade da doença é maior em comparação a crianças mais velhas.^(^^1^^–^^3^^)^Além disso, os potenciais prejuízos e o prognóstico da COVID-19 em lactentes permanecem desconhecidos. A infecção em lactentes apresenta características clínicas/radiológicas distintas em comparação as dos adultos e responde por 48% das internações de todas as crianças e adolescentes infectados.^(^^2^^)^As manifestações clínicas relatadas em lactentes infectados pelo SARS-CoV-2 são predominantemente respiratórias, como febre, tosse, rinorreia, aumento do trabalho respiratório e letargia. Ainda, quase 25% dos casos também incluem eventos gastrintestinais (GI), como vômitos, diarreia, dor abdominal, intolerância alimentar ou ingesta reduzida.^(^^1^^,^^2^^)^

Quase 50% das crianças infectadas pelo SARS-CoV-2 foram coinfectadas por patógenos respiratórios.^(^^2^^,^^4^^)^Atualmente, a atenção tem se voltado ao trato GI,^(^^5^^)^já que evidências recentes indicam que o SARS-CoV-2 consegue colonizar e se replicar ativamente ali.^(^^1^^,^^6^^)^Esses achados têm importantes implicações na transmissão e no manejo da doença, e na ocorrência de coinfecção enteropatogênica por *Clostridioides difficile* , conhecido anteriormente como *Clostridium difficile* .

Crianças colonizadas com *C. difficile* toxigênico raramente desenvolvem sintomas clínicos, e as razões para isso são desconhecidas.^(^^7^^)^Existem lacunas consideráveis nos dados disponíveis sobre COVID-19 em lactentes e coinfecção por *C. difficile* . Descrevemos aqui um relato de caso de uma lactente com COVID-19 coinfectada por *C. difficile* . Até onde sabemos, não há relatos sobre esse tipo de coinfecção enteropatogênica em recém-nascidos e lactentes.

## RELATO DE CASO

Uma lactente do sexo feminino, 2 meses de idade, previamente saudável, foi recebida no serviço de emergência pediátrica. A mãe relatou perda de peso, cólica, choro por fome alternado com letargia, diarreia leve e piora dos vômitos, com início há 5 dias ( Figura 1 ). Relatou ainda como sintomas anteriores congestão nasal, tosse esporádica, conjuntivite, pequenas vesículas cutâneas no tórax e ausência de febre. O resultado do teste nasofaríngeo de transcrição reversa seguida de reação em cadeia da polimerase (RT-PCR) para SARS-CoV-2 foi positivo ( Tabela 1 ). A mãe se infectara pelo SARS-CoV-2 10 dias antes da criança. Ao exame físico, a criança mostrou-se muito irritável, alternando com letargia, com fácies de dor sugestiva de cólica abdominal associada a múltiplas interrupções da alimentação, desidratação moderada e hipotermia (<36°C). O peso era de 4,8kg; a pele tinha aspecto rendilhado, com enchimento capilar prolongado e diminuição do débito urinário. O pulso era de 125 batimentos/minuto, a frequência respiratória de 54 respirações/minuto, e a saturação de oxigênio na oximetria de pulso era de 97% em ar ambiente. A paciente foi transferida para a unidade de terapia intensiva pediátrica (UTIP) após ressuscitação volêmica.

**Figura 1:** Cronologia de eventos clínicos, laboratoriais e de imagem

**Tabela 1:** Resultados laboratoriais e de imagem durante o curso clínico

Variáveis	Dia da internação	Dia 10	Dia 27
SARS-CoV-2	*Swab* oral: positivo Fezes: positivo		Swab oral: negativo Fezes: positivo
Hemoglobina, g/dL	11,4	9,6	10,5
Hematócrito, %	31,9	27,7	30,5
Leucograma, μL	12.460	9.940	7.970
Neutrófilos, μL (1.000-8.500)	623	2.565	1.363
Linfócitos, μL (4.000-13.500)	9.843	6.706	6.481
Plaquetas, ×10^3^/μL (150-450)	470	503	427
PCR, mg/L (<5,0)	<1	0,3	<0.3
Albumina, g/dL (3,1-5)	4,1		
Ferritina, ng/mL (8,46-580)	503	388	
AST, U/L (0-32)	70		36
ALT, U/L (<25)	42		18
Ureia, mg/dL (4-16)	8		
Creatinina, mg/dL	0,30		
Tempo de protrombina, (16-26,1)	29,6		27,9
Fibrinogênio, mg/dL (200-400)	177		161
TTPa, (25,6-35,5)			33,5
Dímero-D, ug/mL (<500)	641	751	
Troponina, pg/mL (<5)	11	22	16
IL-6, pg/nL (1,5-7,0)	2,2		
IL-10, pg/nL (0-2)	2,3		
Hemocultura	Negativa		
*Clostridioides difficile,* fezes	Positivo	Positivo	Positivo
Ecocardiograma	Normal	Normal	
Ultrassom transfontanelar			Normal
Ultrassom de abdome	Normal		

SARS-CoV-2: coronavírus da síndrome respiratória aguda grave 2; PCR: proteína C-reativa; AST: aspartato aminotransaminase; ALT: alanina aminotransferase; TTPa: tempo de tromboplastina parcial ativada; IL: interleucina.

A paciente foi uma recém-nascida a termo, sem nenhuma doença preexistente ou em uso de antibiótico, alimentada com leite materno e complementação com fórmula infantil desde a segunda semana de vida.

A tabela 1 resume os dados de exames de bioquímica, urina, fezes, testes de função de coagulação e biomarcadores de infecção, realizados após confirmação da infecção pelo SARS-CoV-2 no momento da internação e no acompanhamento. A sorologia para SARS-CoV-2 foi positiva para IgM (2,11AU/mL, com faixa de normalidade <0,90) e negativa para IgG. O PCR para SARS-CoV-2 PCR permaneceu positivo nas amostras fecais por mais de 27 dias, apesar de o exame do trato respiratório já ter resultado negativo. Testes adicionais de painel viral, cultura de urina e fezes foram negativos, e apenas o teste de amplificação de ácido nucleico (NAAT ou PCR) foi positivo para *C. difficile* .

Tomografia computadorizada (TC) helicoidal *multislice* de tórax, ecocardiograma e ultrassom de abdome foram normais.

Durante 4 dias de internação na UTIP, a paciente persistiu com cólicas abdominais e precisou de quantidades adicionais de fluidos intravenosos, devido à má aceitação do leite materno e da fórmula infantil. O inibidor de bomba de próton inicialmente prescrito foi suspenso. Não foram prescritos antivirais, glicocorticoides ou antimicrobianos para a paciente.

## DISCUSSÃO

Os casos relatados de COVID-19 em lactentes revelam que a manifestação GI pode ser a única apresentação clínica da doença, sendo mais proeminente que os sintomas respiratórios, como aconteceu na paciente descrita.^(^^2^^,^^3^^,^^5^^)^É importante ressaltar que a COVID-19 em lactentes pode ser uma doença mais grave que em crianças mais velhas, devido à imaturidade do sistema imunológico. Consequentemente, os lactentes têm maior risco de complicações mais significativas e podem exigir cuidados intensivos.^(^^2^^,^^8^^)^

Coinfecções com outros patógenos respiratórios, além do SARS-CoV-2, são comuns, mas casos com enteropatógenos e, particularmente, com *C. difficile* , ainda não foram relatados. O espectro clínico da doença por *C. difficile* varia desde diarreia secretória autolimitante até colite pseudomembranosa e choque séptico. Duas características importantes de *C. difficile* em lactentes são a incidência mais alta no primeiro ano de vida (14% a 37%) e casos frequentes de colonização sem sintomas clínicos relevantes.^(^^7^^)^O teste PCR para *C. difficile* detecta o gene regulador (tcdC) responsável pela produção das toxinas A e B, sem detectar as toxinas propriamente ditas, e, frequentemente, os resultados refletem a colonização por *C. difficile* , e não a doença. Por motivos ainda desconhecidos, lactentes colonizados não apresentam efeitos toxigênicos das exotoxinas liberadas por *C. difficile* , diferentemente de crianças mais velhas e adultos, que podem apresentar diarreia e colite graves. Acredita-se que a mucosa intestinal imatura ainda não tenha receptores suficientes para a toxina de *C. difficile* .^(^^7^^)^Outro fator importante é o efeito protetor do leite materno, já que lactentes alimentados com leite materno têm menor taxa de colonização por *C. difficile* (14%) que aqueles alimentados com fórmula infantil (30%). Nesse caso, os danos causados ao intestino pelo SARS-CoV-2 podem facilitar a infecção por *C. difficile* . Apesar dos efeitos protetores do leite materno e da imunidade dos lactentes, isso não foi suficiente para fazer cessar a eliminação do vírus pelas fezes. Além disso, a supressão ácida com inibidor de bomba de próton pode facilitar a infecção por *C. difficile* .^(^^9^^)^

Achados laboratoriais da paciente mostraram pequenas alterações no leucograma, e os marcadores inflamatórios, proteína C-reativa e ferritina estavam normais, conforme relatado em outro estudo semelhante.^(^^3^^)^Entretanto, biomarcadores mais específicos, como alterações no coagulograma, trombocitose, aumento na concentração sérica de IL-10 e dímero-D, foram sugestivos de um certo grau de doença inflamatória sistêmica. O ecocardiograma e a TC de tórax da paciente foram normais. A importância da TC para o diagnóstico da COVID-19 em crianças é questionada por alguns autores, porque metade dos casos não apresentou alterações radiológicas durante o curso da doença.^(^^4^^)^

A paciente não recebeu agentes antivirais, glicocorticoides e nem antimicrobianos, porque a eficácia desses medicamentos é incerta.^(^^10^^)^Incentivamos o aleitamento materno por seus efeitos protetores contra SARS-CoV-2 e *C. difficile* e por este ser uma fonte improvável de transmissão da infecção. Além disso, não há dados suficientes que demonstrem a eficácia dos probióticos no tratamento da infecção por *C. difficile* .

Conforme os achados de outros autores, o exame de fezes da paciente continuou positivo para SARS-CoV-2 1 mês após o diagnóstico, apontando para a possibilidade de transmissão fecal-oral prolongada.^(^^4^^)^

## CONCLUSÃO

Este caso revela que o impacto em longo prazo da COVID-19 na saúde dos lactentes ainda é desconhecido. Muitas incertezas persistem em relação à infecção pelo SARS-CoV-2 em lactentes, principalmente quando coinfectados por outros enteropatógenos. São necessários dados mais robustos e estudos com uma amostra maior de lactentes para esclarecer os determinantes das manifestações gastrintestinais e da gravidade da infecção. O aleitamento materno parece ser um fator de proteção e pode ser mantido em mães infectadas pelo SARS-CoV-2.
